# Wheezing-Related Relevant Factors and the Role of Viral Bronchiolitis

**DOI:** 10.3389/falgy.2021.726972

**Published:** 2021-10-05

**Authors:** Alvaro Teijeiro, R. Maximiliano Gómez

**Affiliations:** ^1^Respiratory Department, Children's Hospital, Córdoba, Argentina; ^2^School of Health Sciences, Catholic University of Salta, Salta, Argentina

**Keywords:** bronchiolitis, wheeze, asthma, childhood, recurrence, risk

## Abstract

Bronchiolitis is a virus-associated infection of the lower respiratory tract exhibiting signs and symptoms of airway obstruction. Respiratory Syncytial Virus (RSV) is responsible in most cases; however, different rhinoviruses have also been implicated. Specific viruses and time until the first infection, severity of the respiratory condition, and atopic status have a determinant role in the recurrence of wheezing and asthma development. Genetics, lung function, atopic condition, the role of microbiota and environment, pollution, and obesity are considered in the present review. Emergency room visits and hospitalizations because of severe wheezing and smoking during pregnancy among others were identified as risk factors for significant morbidity in our population. Approaching determinant conditions like genetics, allergy, antiviral immunity, and environmental exposures such as farm vs. urban and viral virulence provides an opportunity to minimize morbidity of viral illness and asthma in children.

## Introduction

Bronchiolitis is described as the first phase of low respiratory tract infection (LRTI) in children under the age of 2 years with viral etiology and is clinically expressed by peripheral airway obstruction (cough, rales, and/or wheezing) (1). It predominates in autumn-winter months, with Human Respiratory Syncytial Virus (HRSV) being the most constant etiological agent, behind 60–80% of bronchiolitis (2). Data from Argentina reported that HRSV accounted for 81.3% in patients hospitalized for LRI, with a fatality rate of 1.7% (3, 4). New molecular techniques have amplified the knowledge of human rhinoviruses (HRV) A, B, and C which have joined the list of agents responsible for lower respiratory infection, including bronchiolitis (5) and pneumonia (6) besides their role of inducers of wheezing and asthma in childhood (7). Bronchiolitis is much more common between 3 and 5 months of age. Less than 3% of infants without risk factors require hospitalization and among them, mortality is <1%. In Argentina, lower respiratory infections (LRIs) are the third most frequent cause of death in infants within their first year (8), and recent data evidenced its growing incidence in emergency room visits in this population (9).

Recurrent wheezing corresponds to three or more incidents within twelve consecutive months. Mostly, the first event happens within the first year (10). In consequence, identification of risk factors for recurrent and/or persistent wheezing in infants is mandatory.

Significant association between LRTI and wheezing in infants has been reported in the EISL study (11), but also with asthma development (12). We identified the need for emergency room visits because of the perception of severe wheezing by parents, hospitalizations, and frequent night time awakening because of wheezing are variables significantly associated with recurrent wheezing (RW); and smoking during pregnancy, emergency room visits because of severe wheezing, and inhaled corticosteroids prescription were associated with severe wheezing (SW) more than six episodes in the first year of life (13). The presence of bronchiolitis in the first 3 months of life was found to be related to both RW and SW, and having had pneumonia was associated with occasional wheezing (OW) and RW.

Around half of infants hospitalized because of bronchiolitis will develop childhood asthma (14, 15). Asthma is a chronic bronchopulmonary disease characterized by bronchoconstriction and airway inflammation, and is among the top five pediatric conditions in the USA (16). In our population, the prevalence of current asthma symptoms was 16% in 6–7-year-old children (17), and recurrent wheezing was 18.9% (13). While diagnosing asthma in the first year of life is difficult, we have to highlight the need of risk factor identification particularly on those patients suffering bronchiolitis.

**Figure 1:**
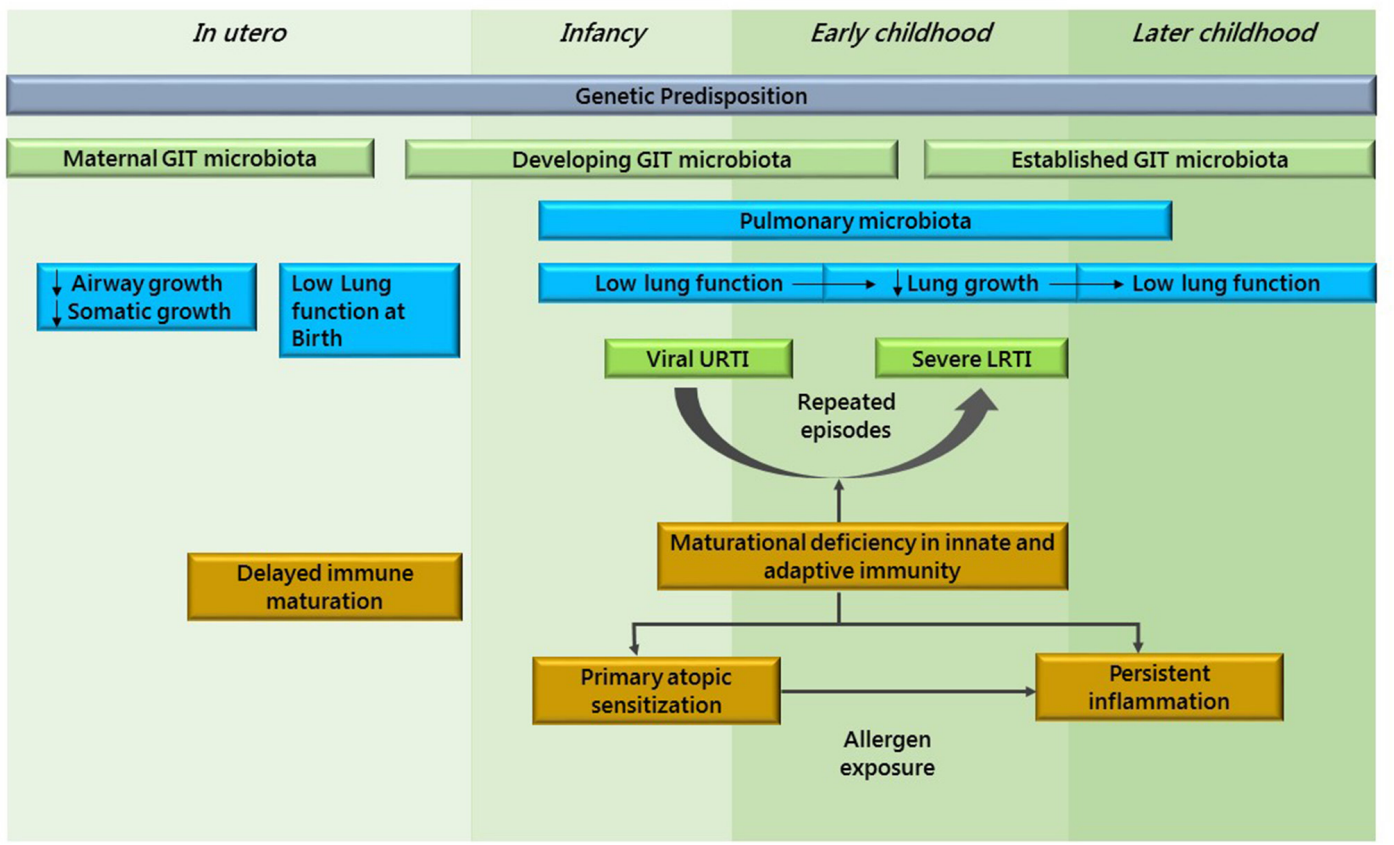
This figure shows asthma as a developmental disease. Adapted from Holgate et al. (23).

New evidence hints that environmental factors may alter genetic background within the first 3 months of life (18, 19). Compared to children born vaginally, those born by C-section have less diverse intestinal microbiota in their first 2 years of life. The development of the immunological system is deeply influenced by intestinal microbiota bacteria, and its alteration has been linked to the development of asthma (20). Not only microbiota but several variables have a role for developing asthma in childhood, such as first degree familiar history of asthma, premature birth, low birth weight, early weaning, few siblings in the household, attending kindergarten, exposure to cigarette smoke during pregnancy, low socioeconomic status, and low parental level of education (21, 22) (Figure 1).

Taking into account that pre-commitment of the airways may underlie infection, different lines of research aiming to identify risk factors in preschool children who develop asthma are considered.

### Viral Infection (RSV and RV) and Atopy

Specific viruses and time until the first infection, severity of the respiratory condition and atopic condition of child are determinants. An early onset wheezing episode induced by RV, RSV, or other viruses increases the risk for asthma at the age of 17–20 years compared to controls without wheezing history (24).

Then, is the bronchiolitis and asthma relationship cause or coincidence? The COAST study linked allergic sensitization within the first year of life with virus-associated wheezing, but the opposite is not true (25). It seems that the two assumptions of bronchiolitis as a cause or as a marker of asthma are not mutually exclusive. RSV infections usually occur in epidemic outbreaks around winter, producing a cytopathic effect on the epithelial airway in children younger than 3 months who characteristically require hospitalization (26). On the opposite side, rhinovirus infections are present throughout the year, involving older outpatient children with a frequent family history of asthma and/or atopy (27, 28). While rhinovirus bronchiolitis appears as a risk marker for asthma and atopy, RSV bronchiolitis may act as a cause, mostly in severe cases requiring hospitalization (29, 30).

The RSV Bronchiolitis in Early Life (RBEL) study reinforces the association between severe RSV bronchiolitis and incidental asthma: in 206 hospitalized infants, half received an asthma diagnosis by the age of 7 years (31).

Rhinovirus A is the most prevalent etiologic virus in acute bronchiolitis; and respiratory viruses such as the rhinovirus family are also associated with both bronchiolitis and the potential development of asthma (32, 33). Other studies show that preschoolers with early viral wheezing who have been referred to a pediatric hospital due to severe symptoms will have a higher risk of asthma between the ages of 5 and 10 (34).

Other studies found that rhinovirus C infection was related to the highest risk of RW, particularly on those with IgE sensitization during infancy. Additionally, these data suggest that RW resulted in asthma at the age of 4 years, while another study showed that rhinovirus infections significantly predicted RW in a group of infants with bronchiolitis under study for 3 years (35).

Bronchial epithelial cells are pre-committed to a type-2 (E2)-like phenotype in atopic patients. Once activated, their epithelial alarmins TSLP, IL-31, CCL-26, IL-25, and IL-33 induce a local inflammatory type-2 response with the characteristic cytokines IL-4, IL-5, IL-9, IL-13, IL-25, IL-33, and the increase of eosinophils (36). Allergen-specific immunotherapy, the most personalized treatment for allergic conditions, induces some kind of local gene expression footprint in the lower airways, which can be explained by multiple regulatory pathways that go beyond the scope of this review (37).

Rhinoviruses affect both airway epithelial cells and macrophages and activate them, with differences in the innate immune response between infants who develop asthma compared to non-atopic children (38). Deficiencies in innate cytokine production (e.g., interferons) were associated with the subsequent risk of wheezing (39). Several reports evidence recurrent wheezing following RSV bronchiolitis, and a significant number of infants with preschool RW will maintain their wheezing condition into adolescence (40).

Additional recent findings describe the role of RV infection in children with acute bronchiolitis, its impact on subsequent asthma development, and the implication in clinical practice (41), while familial history of atopy and RSV-hRV co-infection are risk factors for recurrent bronchial obstruction and sensitization (42).

A different phenotype compared to those who transiently wheeze seems evident. There are several reports correlating viral infection, atopy, and genetic burden. Then, we might consider all these variables.

## Asthma Predictive Index

It is widely proclaimed that ~80% of asthmatic children present with symptoms in the first few years of life, whereas about 30% of early wheezers will develop physician's diagnosed asthma (43). Among the leading representative index is the Asthma Predictive Index (API), which was derived from the Tucson's Children's Respiratory Study (TCRS) (44). According to their established criteria, diagnosis of asthma is predicted by recurrent wheezing episodes more than four times previously plus one of the two major criteria (physician-diagnosed eczema or parental asthma), or two of the three minor criteria (physician-diagnosed allergic rhinitis, wheezing without cold, or peripheral eosinophilia concentration not <4%). Positive API values were highly predictive of current asthma between the ages of 6 and 13 years (as high as 77%), and negative values discarded asthma as a cause in 68% of cases. Specificity is as high as 97%, but its sensitivity was low (15–22%), which is consistent with new asthma in adulthood having had minimal wheezing during childhood. Adopting the “loose index” (at least one wheezing episode during the first 3 years, without changing other criteria), sensitivity would increase up to 57%, although several patients who would not become asthmatic later in life (i.e., transient-early wheezers) may be misclassified as positive, potentially receiving unnecessary treatment (45). Then, API was modified according to the Prevention of Early Asthma in Kids study: more than one inhalant allergen sensitization was added as a major criterion, and food allergen sensitization (milk, eggs, and peanuts) were substituted for physician-diagnosed allergic rhinitis (46). Only the API derived from the TCRS has a supporting publication to date; when it was applied to the Colombian population, it showed a sensitivity of 43% and a specificity of 79% after a 6-year follow up period (47). In consequence, guidelines like the Global Initiative for Asthma recommend consideration of API when controller medication is prescribed in preschool wheezers (48).

Perhaps the greatest risk factor is familiar asthma; then, the role of 17q21 variants in the development of HRV wheezing illnesses during early childhood was evaluated in a study published in 2013, showing the effects of (17q21 with ORMDL3) genotype on having asthma in a subgroup of children with early onset HRV wheezing. These results emphasize the importance of the joint effects of genetic and environmental conditions in the mechanisms underlying complex diseases (49).

### Relationship Between Infection and Lung Microbiota

Colonization of the upper respiratory airways occurs directly after child birth, and quickly develops particular profiles during their first weeks (50–53). Various cross-sectional and case-control studies described differences in respiratory microbial profiles between children with mild, moderate, and severe respiratory syncytial virus infection (54). Early colonization at birth and the development of URT microbiota during the first months of life may directly influence respiratory health (55). Hence this study (56) provided data that quick microbiota maturation is associated with microbiota instability and also with the number of RTIs along the first year. These changing processes could be seen as early as within the first month of age (i.e., before the first RTI episodes). Then, we could link the impact of known important factors, such as birth mode, feeding regimes, siblings in family, early day care attendance, and microbiota alteration because of antibiotics with susceptibility to RTIs.

Searching for which specific microbes may provide protection has allowed us to identify wide families of species within microbial taxa that may explain the effect of the farming environment. Badellino et al. observed that among Argentinean teenagers, current rural residence and rural residence for over 5 years resulted in diminished odds of current wheezing and allergic rhino-conjunctivitis. These potential protective effects on the presence of atopy and asthma may be explained by “the farm protective effect” (57). While exposure to cowsheds and consumption of unprocessed cow's milk seems to be the main pillars for protection, the diversity of exposure (across gut, airways, and/or skin) and across multiple populations, altogether seems to contribute to the whole effect (58).

### Relationship of Wheezing With Lung Function and Airway Hyper-Responsiveness

Few studies have evaluated lung function in young children (maximal forced expiratory flow at functional residual capacity [V'max FRC]) by rapid thoracic compression in the TCRS (59) and sRaw by the forced oscillation technique in the MAAS (60), while many studies have not evaluated lung function until children reached school-age. V'max FRC was found to be reduced in recurrent wheezers who started wheezing within the first year of life, and when measured before 6 months of age wheezing was reduced only in transient-early but not in persistent wheezers. A progressive decrease in lung function seems to precede the onset of wheezing, and may not be entirely attributable to a sequelae of respiratory diseases. However, this needs to be confirmed by prospective cohort studies.

While transient-early wheezers (but not late-onset wheezers) display decreased lung function early in age, late-onset wheezers (but not transient-early wheezers) have increased airway hyper-responsiveness. And in persistent wheezers, lung function tends to decrease as measured after the age of 6 years, which is indicative of airway remodeling, a long-term unwanted complication in asthmatic patients. *In utero* and early life, conditions must be addressed in order to gain confidence around these statements (61, 62).

### Gender and Familiar Influence

Having maternal asthma history evidenced a risk factor for wheezing in boys (OR = 1.79, 1.29–2.48, *p* = 0.0004) and girls (OR = 2.05, 95% CI 1.44–2.91, *p* < 0.0001), with a similar effect on wheezing throughout the years. Paternal asthma (OR = 1.83, 1.38–2.57, *p* = 0.0005) and infant bronchiolitis (OR = 2.15, 1.47–3.14, *p* < 0.0001) have been associated with boys only, with no differences at all ages (63). Another Canadian study coordinated by Tse et al. described that maternal asthma, infant bronchiolitis, and atopic dermatitis were all significantly linked with persistent wheezing in both genders, however paternal asthma was a risk factor for persistent wheezing in boys only (OR = 4.27, 95% CI 2.33, 7.83, *p* for sex by paternal asthma interaction = 0.02), while being Black or Hispanic was associated with wheezing only in girls (64).

### Air Pollution

Indoor and outdoor pollution has increased side by side with urbanization and population growth and has become an important contributing factor for the incidence and exacerbation of asthma, with particular emphasis in the developing world (65, 66). Pollutants associated with asthma exacerbations are ozone, nitrogen dioxide, volatile organic compounds, particulate matter (PM10 and PM2.5), and traffic-related air pollution, containing vehicles exhaust fumes and non-combustion particles. These pollutants provoke epithelial damage, activate oxidative stress with consequent airway inflammation (67, 68), and reduce inhibitory T-Reg function (69). These immune inflammatory mechanisms have been proposed to influence asthma development and contribute to augmented respiratory sensitization to inhalant allergens (70) and enhanced airway hyper-responsiveness and remodeling (71). It has also been described that exposure to pollutants prenatally might confer risk to development of postnatal asthma (72), interacting with glutathione S-transferases, and promoting TNF at a genetic level as well (73), influencing gene function through the promotion of methylation and histone acetylation (74).

#### Tobacco Smoke

As a result of mixed compounds including nitrogen dioxide and several volatile organic elements, tobacco smoke has been implicated as a remarkable independent risk factor for asthma incidence (75, 76) partially acting as a T2-type response enhancer (77). Also, grand-maternal smoking during pregnancy elevated the risk for asthma development over the second generation (78) through intrauterine epigenetic mechanisms involved in asthma causation. In our study we found that SHS was significantly related to more frequent wheezing, particularly when the mother was a smoker (OR = 2.7; IC 95%: 1.4–5.18; *p* = 0.0009) and if she smoked during pregnancy (OR = 4; IC 95%: 1.8–8.5; *p* = 0.0001). In our children, second-hand tobacco smoke was related to the need for emergency room visits because of wheezing (40.87%, *p* = 0.0056), representing a significant risk factor for the presence and severity of it (79).

### Obesity

Obesity is strongly associated with the incidence and severity of asthma (80) but also with maternal obesity history, and elevated gestational weight gain was associated with an increased risk of asthma in children, particularly in non-asthmatic mothers (81). Two cohort studies, Tucson and ALSPAC, have found an association between obesity and asthma in adults when they had asthma at 8 years (82, 83). A combination of minimal persistent inflammation, mechanical, metabolic, and hormonal factors might explain these associations. A chronic inflammatory pathway was linked with obesity, presenting increased levels of inflammatory cytokines, like TNF, IL-1, and IL-6 (produced by macrophages) and adipokines like leptin, chemerin, and adiponectin (produced by adipocytes), altogether inhibiting cell proliferation and causing tissue damage. Leptin has a key role on the differentiation of lipo-fibroblasts and controls pulmonary surfactant synthesis in fetal lungs (84). In mouse models, leptin infusion increased airway hyper-responsiveness, enhanced IgE levels as a result of allergen challenge (85), and elevated levels of IL-6 in the airways (86). Elevated maternal levels of leptin favor a low-grade inflammatory status, contributing to the postnatal risk of asthma (87).

However, the exact mechanism linking obesity and asthma still remains to be fully defined.

### Virome

Nowadays, multiple omic and molecular techniques such as metagenomic DNA sequencing is unveiling the virome in human airways. In asthmatic airways, particular microbial colonization may condition not only asthma development but also exacerbations (88).

Anelloviruses (AVs) are present during early life and seemingly do not cause harm to the host. Even though their role in the development of asthma is elusive, an increase in viral load has been implicated in acute respiratory disease, exacerbated chronic lung disease, lower CD3+ and CD4+ T cell numbers, and higher B cell and eosinophilic counts (89, 90).

Besides AVs, Caudovirales and Picornaviridae have been described in asthmatic children. Nevertheless, equilibrium of this core virome and bacteriophages seems to be necessary against asthma symptoms (91). More research in the field is mandatory.

## Conclusions and Future Perspectives

**Figure 2:**
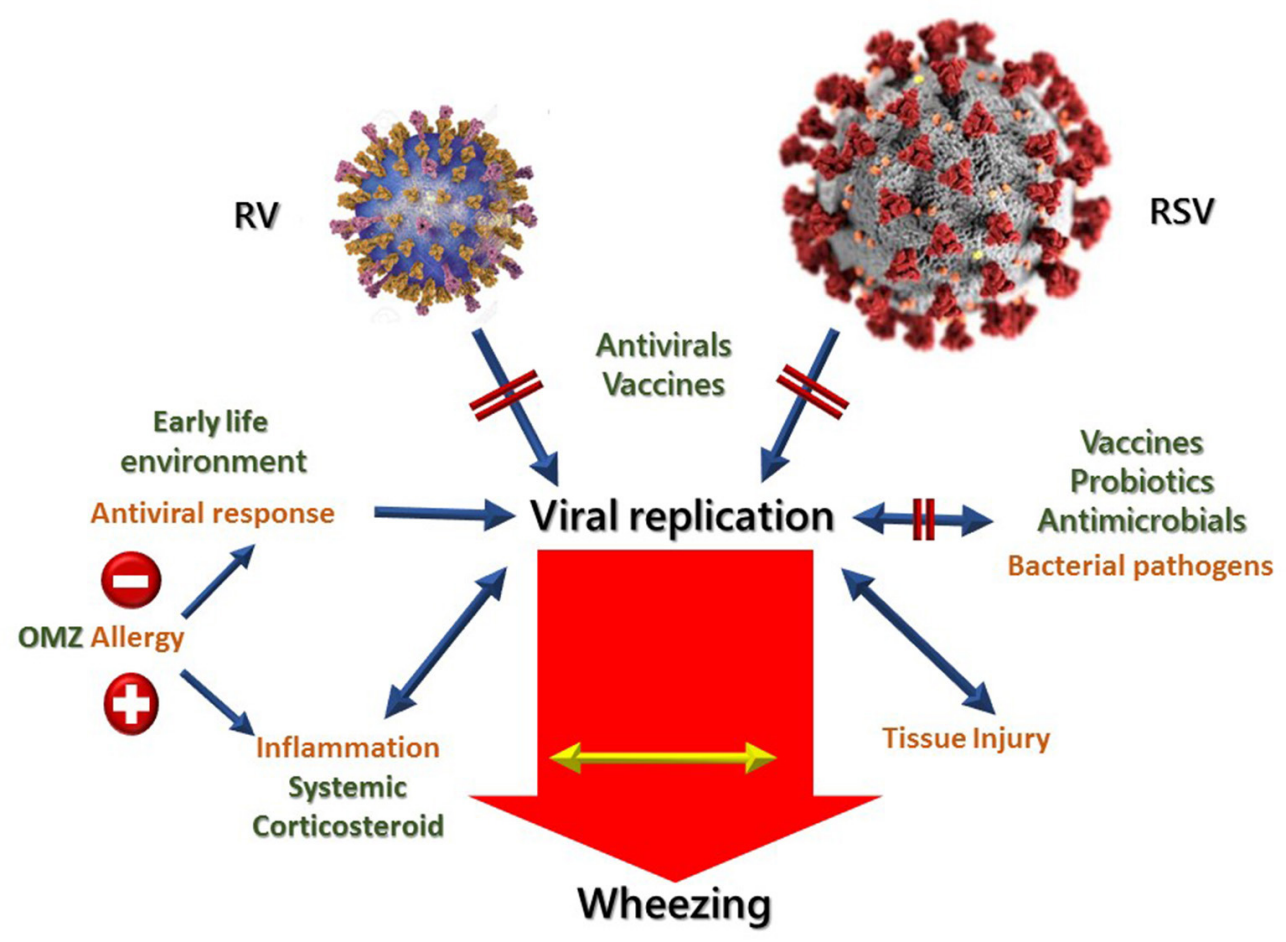
Possibilities of treating and preventing viral induced wheezing diseases. Opportunities for intervention are marked in red. OMZ, Omalizumab. Adapted from Jartti et al. (92).

Several interventional options have emerged to control the recurrence and severity of viral respiratory diseases, and hopefully the incidence and exacerbations of asthma (Figure 2).

The search should focus on effective and safe antivirals and vaccines for RV and RSV, where candidates are currently being investigated in clinical trials (93). Atopic condition has been described both as a frequent condition and also as a risk factor for asthma and wheezing. Through blocking lgE with monoclonal antibody omalizumab, it is possible to ablate antigen signaling through IgE-related mechanisms and re-establish IFN-a response to rhinovirus, reducing asthma exacerbations in viral season as well. In this regard, the PARK study is an ongoing double-blind, placebo-controlled trial with omalizumab in 2–3-year-old infants with a high risk of asthma (94).

Another strategy under evaluation in high-risk young children, in order to prevent wheezing condition and asthma incidence, is the oral administration of bacterial lysates (95).

Finally, therapeutical interventions able to reduce or stop allergies may strengthen antiviral pathways with consequent diminished inflammatory mechanisms responsible for airway obstruction and the remodeling process. It is mandatory to establish a global approach involving personal risk factors like genetics, allergic sensitization, and innate immune system status, together with environmental pollutants and microbiota, in order to lessen viral respiratory morbidity and childhood asthma.

## Author Contributions

All authors listed have made a substantial, direct and intellectual contribution to the work, and approved it for publication.

## Conflict of Interest

The authors declare that the research was conducted in the absence of any commercial or financial relationships that could be construed as a potential conflict of interest.

## Publisher's Note

All claims expressed in this article are solely those of the authors and do not necessarily represent those of their affiliated organizations, or those of the publisher, the editors and the reviewers. Any product that may be evaluated in this article, or claim that may be made by its manufacturer, is not guaranteed or endorsed by the publisher.
